# A bibliometric analysis and visualization of literature on non-fasting lipid research from 2012 to 2022

**DOI:** 10.3389/fendo.2023.1136048

**Published:** 2023-04-19

**Authors:** Yilin Hou, Zehua An, Xiaoyu Hou, Yunpeng Guan, Guangyao Song

**Affiliations:** ^1^ Graduate School, Hebei Medical University, Shijiazhuang, Hebei, China; ^2^ Department of Endocrinology, Hebei General Hospital, Shijiazhuang, Hebei, China; ^3^ Department of Rehabilitation, Hebei General Hospital, Shijiazhuang, Hebei, China; ^4^ Department of Internal Medicine, Hebei Medical University, Shijiazhuang, Hebei, China

**Keywords:** non-fasting lipid, postprandial lipid, cardiovascular diseases, diabetes, oral fat tolerance test, bibliometric analysis, VOSviewer, citespace

## Abstract

**Background:**

Non-fasting lipid assessment can help predict cardiovascular disease risks and is linked to multiple diseases, particularly diabetes. The significance of non-fasting lipid levels in routine screening and postprandial lipid tests for potential dyslipidemia has not been conclusively determined. Various new lipid-lowering strategies have been developed to improve non-fasting dyslipidemia. Therefore, analysis of scientific outputs over the past decade is essential to reveal trends, hotspots, and frontier areas for future research in this field.

**Methods:**

The Science Citation Index Expanded in the Web of Science Core Collection database was searched for publications related to non-fasting lipid research from 2012 to 2022. The regional distributions, authors, disciplines, journals, references, and keywords of the studies were analyzed using the bibliometric software VOSviewer and CiteSpace.

**Results:**

A total of 4160 articles and reviews that met the inclusion criteria were included in this study. The output trend was established to be stable and the number of citation indices has been persistently increasing. A total of 104 countries/regions, 4668 organizations, and 20782 authors were involved in this research area. In terms of country, the United States had the largest number of publications (979). The University of Copenhagen was the most productive institution, publishing 148 papers. Professor Børge G Nordestgaard has made the most significant contribution to this field. *Nutrients* was the most productive journal while the *American Journal of Clinical Nutrition* was the highest co-cited journal. Analysis of co-cited references indicated that lipid-lowering strategies, statin therapy, high-fat meals, insulin resistance, physical exercise, and fructose were hotspots. Analysis of co-cited keywords revealed that apolipoprotein B, especially apolipoprotein B48, is becoming a key research focus. The keywords “gut microbiota” and “meal timing” were the most extensively studied.

**Conclusion:**

The causal relationship between non-fasting dyslipidemia and diseases is currently being explored and the standards for non-fasting or postprandial lipid assessment are continuously being updated. Among the hotspots, lipid-lowering strategies are a potential research direction. Apolipoprotein B48, gut microbiota, and chrononutrition are the research frontiers. This initial bibliometric analysis of non-fasting lipids will enable researchers to monitor swift transformations and recognize novel concepts for upcoming research.

## 1 Introduction

Lipid profile standard measurements include triglycerides (TG) and cholesterol. They also include lipoprotein (a), apolipoprotein B (Apo B), apolipoprotein A1 (Apo A1), lipoprotein subfractions, other apolipoproteins, and metabolomics phenotyping in expanded or additional lipid profiles (1). Guidelines and consensus statements from nearly ten years ago mandated that lipid profile samples be collected after a period of fasting. Study subjects were required to fast overnight for at least 8 h before blood samples were drawn. However, compared to fasting TG, non-fasting TG performed better in cardiovascular risk prediction (2). Evidence from prospective cohort studies based on large populations revealed that elevated non-fasting TG levels are independently associated with increased risks of myocardial infarctions, ischemic heart disease, death (3), total cardiovascular events (2), and ischemic stroke (4). Furthermore, blood sampling in the non-fasting state was convenient for both laboratory personnel and patients (5). In 2009, the Danish Society of Clinical Biochemistry was the first society to recommend the testing of lipid profiles in the non-fasting state to predict cardiovascular risk (6). Currently, the debate on fasting and non-fasting lipid measurements is ongoing. Societies, guidelines, and statements from the United States (7–9), Europe (10, 11), Canada (12), Brazil (13), and India (14) recommend routine screening via non-fasting lipid measurements.

The fasting state only occurs in a short period before breakfast, which does not reflect the real lipid concentration throughout a 24-hour day (5). Intestinal-derived lipoproteins, especially postprandial triglyceride-rich lipoproteins (TRL), can be assessed in blood samples after meals as supplementary to liver-origin lipoproteins in fasting samples. Among the lipid profiles, TG levels fluctuate with different meals and meal times. Immensely high TG levels interfere with estimation of LDL-C levels (15). Therefore, establishing optimal cut-offs for non-fasting lipid profiles is a major challenge in clinical practice. Postprandial TG concentration, which can be detected by an oral fat tolerance test (OFTT), is also a cardiometabolic risk factor (16). The OFTT standards have not been fully established (10).

Non-fasting lipid metabolism is not only associated with cardiovascular disease (CVD), but also with diabetes (17), metabolic syndromes (18), non-alcoholic fatty liver disease (19), and polycystic ovarian syndrome (20) among others. The pathophysiological processes associated with postprandial metabolic responses have a connection with insulin resistance (21), oxidative stress (22), endothelial dysfunctions (23), and gut microbiota (24) among others. Nutrition (25), physical exercise and medication (26) play an important role in prevention and treatment strategies. Given the high number of studies, it is important to summarize the worldwide status, trends, and major topics in non-fasting lipid research.

Bibliometrics is a cross-disciplinary technique applied to the quantitative and qualitative analysis of knowledge. It can reveal trends and hotspots by countries/regions, institutions, researchers, keywords, and references based on publications in a field. In this study, we used bibliometrics software to analyze the characteristics of emerging trends and directions for future research on non-fasting lipids.

## 2 Methods

### 2.1 Literature search and data collection

**Figure 1:**
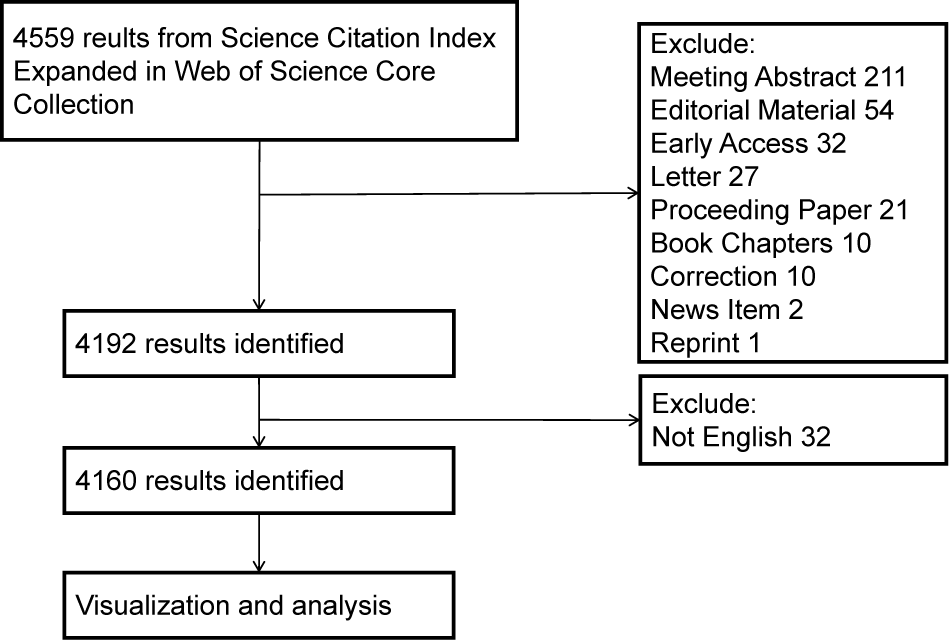
Retrieval and screening process flowchart. CiteSpace allows efficient evaluation of network visualization, while the VOSviewer provides clearer, user-friendly visualization.

We searched the Science Citation Index Expanded 1900-present (SCI-E) of Web of Science Core Collection (WoSCC) from 1^st^ January 2012 to 1^st^ November 2022. This database contains impactful global academic publications, and the downloadable data information is suitable with the bibliometrics software. The search strategy was composed of the following topic words with MESH Unique ID and free words: (“Lipids (D008055)” OR “Hyperlipidemias (D006949)” OR “Triglycerides (D014280)” OR “Hypertriglyceridemia (D015228)” OR “Cholesterol (D002784)” OR “Hypercholesterolemia (D006937)”) AND (“Postprandial period (D019518)” OR “non-fasting” OR “nonfasting”). The articles and reviews written in the English language were included in this study. Data were collected as plain text files with full records and cited references. The data acquisition flow chart is shown in **Figure 1**.

### 2.2 Data analysis and visualization

VOSviewer (1.6.18 version), CiteSpace (6.1.R4 basic version) and Microsoft Excel (2019 version) were used for bibliometric analyses and visualization. VOSviewer is a computer program supported by the Centre of Science at Leiden University in the Netherlands. VOSviewer was utilized to conduct co-authorship analysis, co-occurrence analysis, and bibliographic coupling, followed by visualizing intellectual structure (27). CiteSpace, created by Chaomei Chen at Drexel University in the United States, is a Java-based application (28). Evolution analysis was performed to investigate the development process of the research based on an understanding of its structure using CiteSpace. VOSviewer and CiteSpace use colorful nodes and links to display the characteristics and connections using information extracted from publications. Node sizes refer to the occurrences of a trait. The color of the nodes and connections depends on the cluster. Different clusters are diversely colorized.

First, we summarized the annual publications and citations and built a graph using Microsoft Excel. Second, we used VOSviewer to create a knowledge map based on co-authorship of countries/regions and organizations. The global geographical distribution of publications was established on www.mapchart.net. Third, the networks of authors and co-cited authors were built by VOSviewer. Author productivity and impact are measured by the H-Index in Web of Science. The H-index is defined by the number of publications *h*, where each publication has at least *h* citations (29). Fourth, the research areas summary was downloaded from the literature search website. The dual-map overlay of journals was created by CiteSpace. Fifth, bibliographic coupling journals and co-cited journals visualization were achieved using VOSviewer. Sixth, analysis of co-cited references by CiteSpace provided structural metrics, such as betweenness centrality (BC), citation burst, and cluster mapping (30). Nodes with BC (> 0.10) represented significant connections between clusters. To identify emerging hotspots, burst detection was conducted for a specific period. Burst strength can reflect the influence of an article or a keyword over time. Cluster view of the co-citation network automatically acquired the cluster labels by using “All in One” function of CiteSpace. The Modularity Q value and Sihouette S value were important in interpreting the cluster network. The Q value range was [0, 1]. A clustering modularization was considered significant when the Q value exceeded 0.3. The cluster network was considered better with elevated Q value. The S value range was [-1, 1]. When the S value is close to 1, the cluster is isolated from others. When the S value surmounted 0.3, 0.5, or 0.7, the cluster network was considered homogenous, reasonable, and highly credible. Finally, author keywords for each publication were extracted to create a timeline view of cluster networks and show the strongest citation bursts using CiteSpace. The number of publications can provide a general estimate of the amount of work produced by a team or facility, while citation rates are commonly considered as measures of a paper’s quality, relevance, or interest, indicating the research’s impact. Bibliometric maps provide a great deal of detail. Exploring fields and comparing maps to users’ own expertise should enable users to access this underlying information. A cluster of research specialties is often identified. The map is used to visualize the relationship between countries and research fields (subfields). Co-citations provide a forward-looking assessment of document similarity. Citations can still vary over time due to changes in academic fields. A co-word analysis is used to identify a research field’s structure based on keyword co-occurrences in publications.

## 3 Results

### 3.1 Annual trend of publications and citations

**Figure 2:**
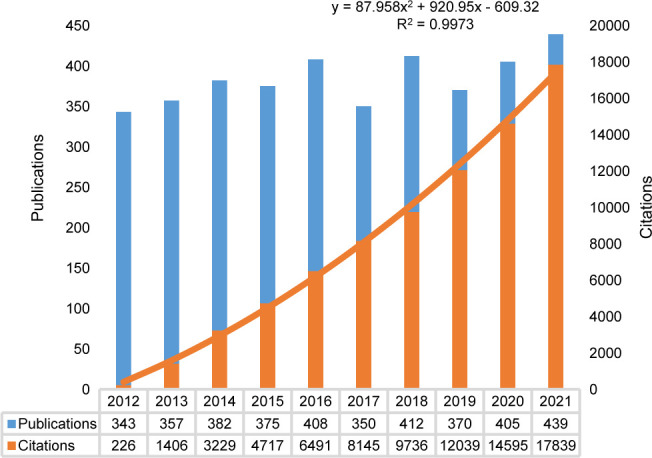
Annual trend of publications and citations. The orange line showed a regression model to illustrate citations per year (2012-2021). A total of 319 publications and 13576 citations were identified from January to November 2022.

The annual distribution of publications and citations is shown in **Figure 2**. Based on our search strategy, we identified 4,160 papers (3,599 articles and 561 reviews), with no duplicates. In the past decade, the number of outputs in the non-fasting lipid field stably fluctuated between 300 and 450 per year. The peak of publications was in 2021, with 439 papers. The H index of this field, as reported by WoSCC (SCIE), was 110 and the average citation per item was 22.09. A regression model [y = 87.958x^2 ^+ 920.95x - 609.32, R² = 0.9973] was adopted to fit the citation over time (2012–2021). 319 publications and 13576 citations from January to November 2022. The findings indicate that there is increasing attention being paid to the field of lipid research that is not limited to fasting conditions.

### 3.2 Distribution of countries/regions and institutions

**Figure 3:**
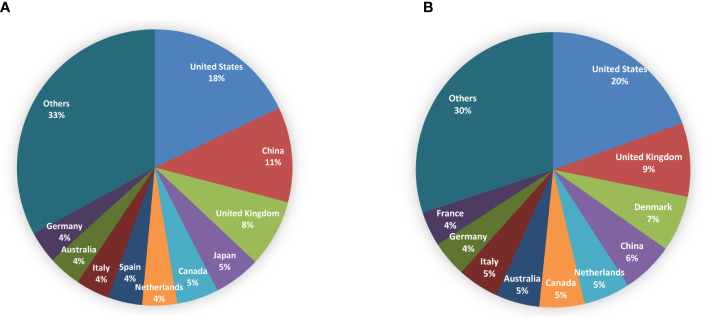
Pie charts for top 10 countries/regions contributing to publications **(A)** and citations **(B)** in the field of non-fasting lipid.

The obtained papers in the non-fasting lipid field were from 104 countries or regions. The top 10 countries/regions contributing to publications and citations were shown in **Figure 3**. The world map of publication distribution was shown in **Supplementary Figure S1A**. Almost half of the output was papers from the United States (979, 18.38%), China (608, 11.41%), the United Kingdom (432, 8.11%), Japan (301, 5.65%), and Canada (267, 5.01%). Publications from the United States (27957, 19.50%), the United Kingdom (12432, 8.67%), Denmark (9411, 6.56%), and China (8757, 6.11%) received the most citations. Regarding the average citation per item, the highest was Italy (30.49), followed by Canada (30.36), the United States (27.13), Netherlands (26.93), and Australia (26.8). Denmark, which had 193 outputs, ranked 11^th^ among the productive countries and had 48.76 average citations per paper. Thirty countries published more than 30 papers. Co-authorship countries/regions network was as shown in **Supplementary Figure S1B**. Studies are active throughout countries in Western Europe, North America, Asia, Oceania, and South America. Cooperation among countries was found to be comprehensive (for instance, China collaborated actively with the United States, the United Kingdom, Canada, Denmark, and the Netherlands).

The analysis revealed 4668 organizations which were engaged in non-fasting lipid research. In **Table 1**, among the top 10 institutions, the University of Copenhagen ranked first in terms of the number of papers (148) and citations (8295). A total of 63 institutions published at least 20 papers in this field. The co-authorship institution network was as presented in **Supplementary Figure S1C**. European institutions, such as the University of Copenhagen, Maastricht University, and Instituto de Salud Carlos III had close cooperation with the University of Toronto and Harvard Medical School in North America, Shanghai Jiao Tong University and National University of Singapore in Asia, University of Western Australia in Oceania, as well as the University of São Paulo in South America.

**Table 1:** Top 10 institutions engaged in research on non-fasting lipid.

Rank	Institutions	Country	Publications	Citations	Citations per Document
1	University of Copenhagen	Denmark	148	8295	56.05
2	Maastricht University	Netherlands	64	1647	25.73
3	University of Toronto	Canada	59	2331	39.51
4	University of California, Davis	USA	57	1696	29.75
5	Instituto de Salud Carlos III	Spain	52	1583	30.44
6	Tufts University	USA	42	1375	32.74
7	French National Institute for Agricultural Research	France	41	1508	36.78
8	King’s College London	United Kingdom	40	1693	42.33
9	University of São Paulo	Brazil	40	866	21.65
10	University of Helsinki	Finland	39	1995	51.15

### 3.3 Authors and co-cited authors

**Figure 4:**
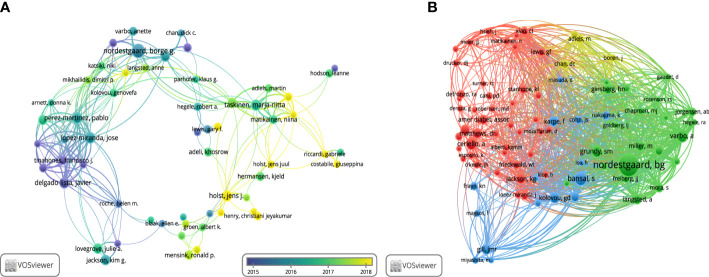
Visualization of authors. **(A)** Collaboration network of co-authors. **(B)** Network visualization of co-citation authors.

Globally, 20782 authors were involved in research on non-fasting lipid. The top 10 productive and co-cited authors, most of whom were from Europe, are shown in **Table 2**. Børge G Nordestgaard at the University of Copenhagen in Denmark ranked first with 43 publications. Out of the authors who published more than 10 papers in this particular field, a total of 89 authors were identified, and 58 of them had formed a collaboration network (**Figure 4A**). Børge G Nordestgaard closely cooperated with José López-Miranda, Pablo Perez-Martinez, and Marja-Riitta Taskinen among others. In **Figure 4B**, Børge G Nordestgaard was the highest co-cited author, with 1144 co-citations. Eighty-four authors were co-cited at least 100 times. Scott M Grundy, Jens Juul Holst, Børge G Nordestgaard, and Marja-Riitta Taskinen each had an H-index higher than 100, suggesting their contributions were significant.

**Table 2:** Top 10 productive authors and co-citation authors in the research field of non-fasting lipid.

Rank	Authors	Publications	H-index	Co-cited Authors	Co-citations	H-index
1	Børge G Nordestgaard	43	144	Børge G Nordestgaard	1144	144
2	José López-Miranda	27	60	Sandeep Bansal	479	20
3	Pablo Perez-Martinez	26	48	Anette Varbo	395	36
4	Jens Juul Holst	25	159	Scott M Grundy	382	166
5	Marja-Riitta Taskinen	24	105	Antonio Ceriello	378	93
6	Javier Delgado-Lista	23	38	Genovefa D Kolovou	321	37
7	Jan Borén	22	66	Jason M.R. Gill	300	44
8	Julie A Lovegrove	22	51	Kim G Jackson	277	29
9	Kim G Jackson	21	29	David R Matthews	276	90
10	Gerald F Watts	21	89	Anne Langsted	272	32

### 3.4 Distribution of disciplines

**Figure 5:**
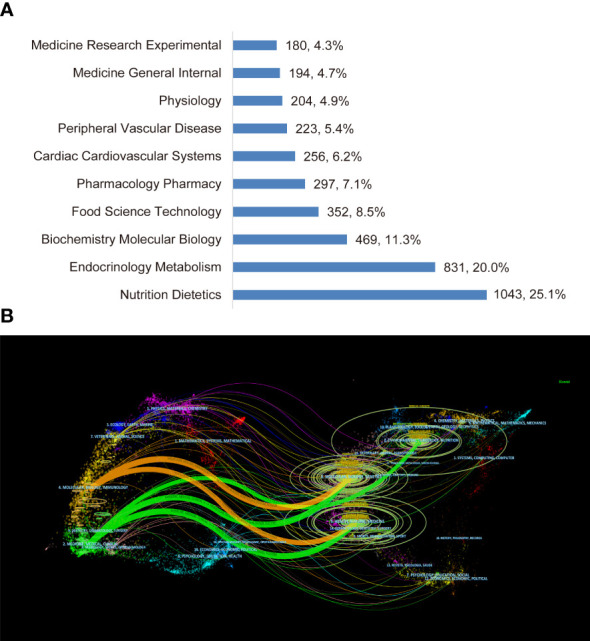
Visualization of disciplines. **(A)** The top 10 disciplines. **(B) **The dual-map overlay of journals.

The top 10 disciplines of non-fasting lipid research in WoSCC (SCI-E) are categorized in **Figure 5A**. Most of the papers referred to the disciplines of Nutrition Dietetics, followed by Endocrinology Metabolism, Biochemistry Molecular Biology, Food Science Technology, and Pharmacology Pharmacy among others.

In the dual-map overlay of journals (**Figure 5B**), major associations between disciplines of citing and cited journals are presented by the orange and green curves. The orange curve represents papers belonging to molecular/biology/genetics and health/nursing/medicine that are cited by molecular/biology/genetics. The green curve represents research output belonging to molecular/biology/genetics, health/nursing/medicine and environmental/toxicology/nutrition that are cited by medicine/medical/clinical researchers.

### 3.5 Journals and co-cited journals

**Figure 6:**
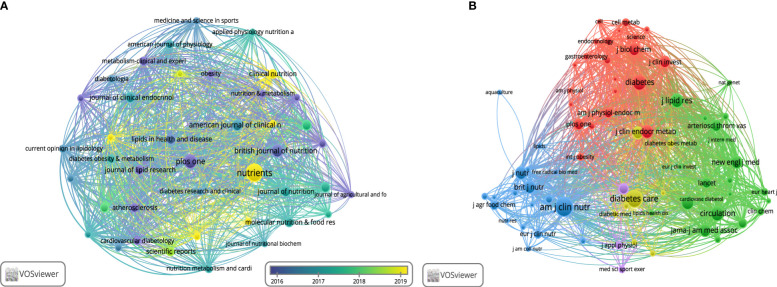
Visualization of journals. **(A)** Bibliographic coupling journals. **(B)** Co-citation journals.

Among the most productive journals, *Nutrients* (169, 4.1%) had the largest output, followed by *Plos One* (104, 2.5%), *British Journal of Nutrition* (84, 2%), *American Journal of Clinical Nutrition* (66, 1.6%), *Journal of Clinical Endocrinology & Metabolism* (59, 1.4%), and *Journal of Nutrition* (58, 1.4%) among others. Of note, 41 of the 940 journals published more than 20 papers in the non-fasting lipid field. The bibliographic coupling network of journals is shown in **Figure 6A**. The network of the co-cited journals is shown in **Figure 6B**. The *American Journal of Clinical Nutrition*, which was co-cited 6501 times, ranked first, followed by *Diabetes Care* (5272), *Diabetes* (4377), *Circulation* (4170), *Journal of Lipid Research* (3677), *Atherosclerosis* (3570), *Journal of Clinical Endocrinology & Metabolism* (3238), and *Journal of Nutrition* (3159). The *New England Journal of Medicine* and *JAMA-Journal of the American Medical Association* had the highest impact factors of 176.082 and 157.375, according to JCR 2021.

### 3.6 Co-cited references and references bursts

Co-cited references analysis was performed using CiteSpace, with setting parameters: timeline from 2012 to 2022, 1 year per slice, node type of references, and selection criteria g index (k = 5). We established a 245-node 1204-link network (**Supplementary Figure S2A**). The nodes surrounded by purple circles had relatively high BC, and were considered key turning points in the non-fasting lipid research field. Guidelines from European Atherosclerosis Society (EAS) about lipids (Chapman MJ, 2011; BC = 0.22) had the highest BC (31), followed by results from the Copenhagen City Heart Study (Nordestgaard BG, 2007; BC = 0.21) (3), a meta-analysis about diabetes and CVD (Sarwar N, 2010; BC = 0.12) (32), and a clinical trial about fructose and fat (Stanhope KL, 2009; BC = 0.11) (33).

**Figure 7:**
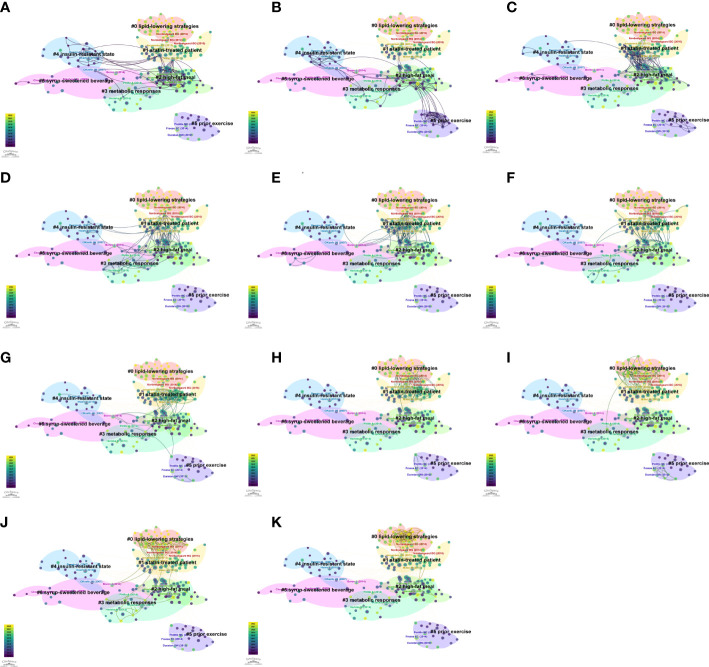
The annual trends of co-citation references network. **(A)** 2012. **(B)** 2013. **(C)** 2014. **(D)** 2015. **(E)** 2016. **(F)** 2017. **(G)** 2018. **(H)** 2019. **(I)** 2020. **(J)** 2021. **(K)** 2022.

We used the “All in One” button to automatically cluster and label the co-cited references. The network, consisting of 7 major clusters (**Supplementary Figure S2B**), was significant with a Q value of 0.472, and highly reliable with an S value of 0.832. From cluster #0 to #6, the number of articles in each cluster declined. The largest cluster #0 lipid-lowering strategies (n = 41, S = 0.814), followed by cluster #1 statin-treated patient (n = 40, S = 0.804), cluster #2 high-fat meal (n = 40, S = 0.67), cluster #3 metabolic response (n = 24, S = 0.901), cluster #4 insulin-resistant state (n = 23, S = 0.941), cluster #5 prior exercise (n = 19, S = 0.977), and cluster #6 syrup-sweetened beverage (n = 17, S = 0.914). The annual transformation of co-cited references is presented in **Figure 7**.

The burst detection function was run to screen citation bursts (**Supplementary Figure S2C**). The articles with the most powerful burst strengths were two prospective follow-up studies published in *JAMA-Journal of the American Medical Association* in 2007. One with a burst of 69.82 has been mentioned above (Nordestgaard BG, 2007) (3), the other with a burst of 60.04 reported on the association between non-fasting TG levels and cardiovascular events (Bansal S, 2007) (2). Review of TRL and a statement about determination of lipid profiles in non-fasting state (Nordestgaard BG, 2016) (34, 35) have received continuous attention from 2017 to 2022. Recent studies by Bhatt DL et al. (36), Ference BA et al. (37), Anderson TJ et al. (12), Mach F et al. (15), Ference BA et al. (38), Langsted A et al. (5), and Grundy SM et al. (7) have achieved significance.

### 3.7 Keyword evolution and bursts

**Figure 8:**
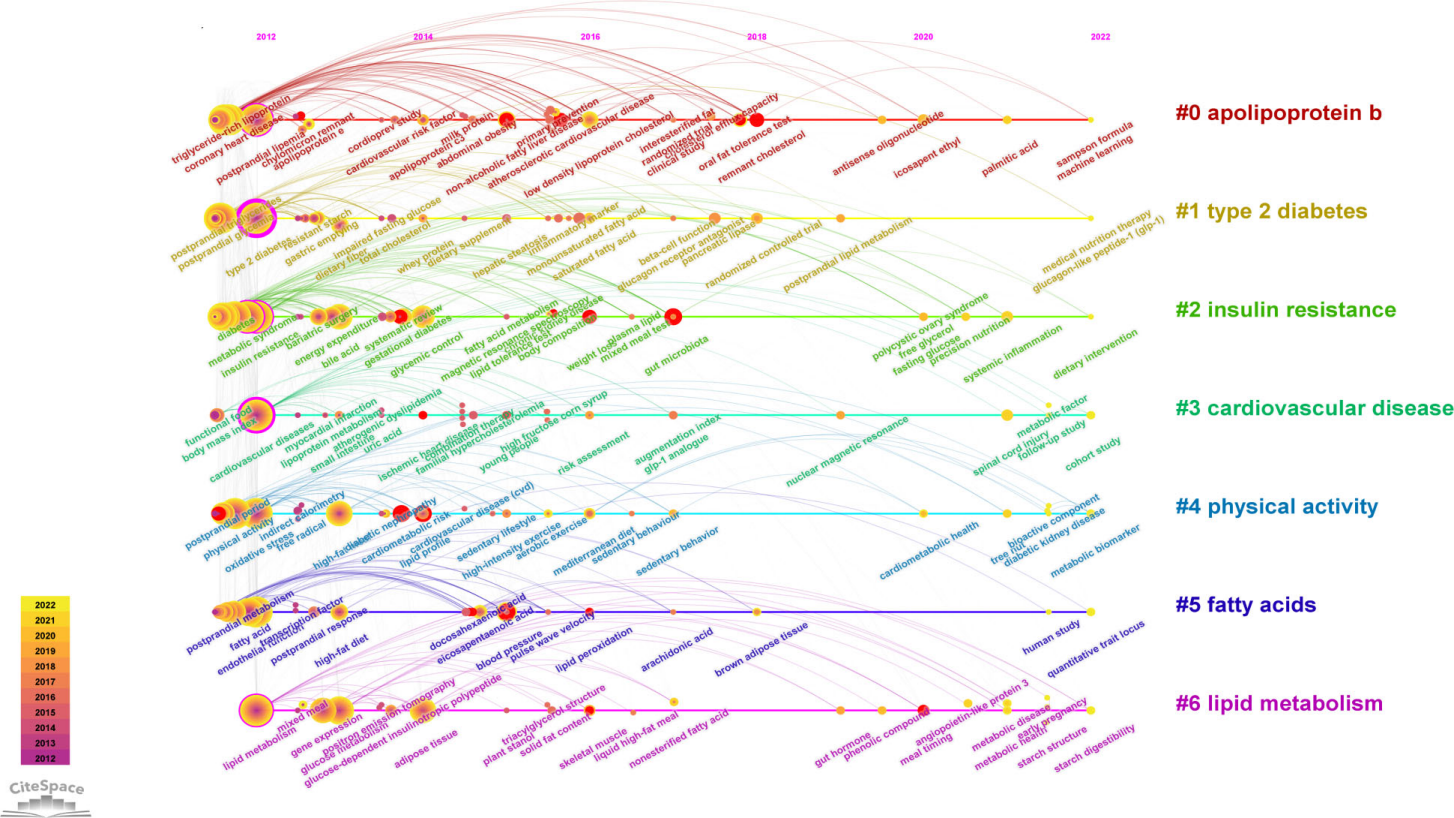
Timeline view of co-citation keywords.

Author keywords reflect the most crucial information in articles. These keywords were analyzed using CiteSpace with parameters; from 2012 to 2022, 1 year per slice, node types of keywords, with the selection criteria as the top 50 per slice. The “All in One” function was used to automatically recognize the clusters and labels. We established a 279-node 1283-link cluster network in **Supplementary Figure S3A**. The timeline view of author keywords revealed the evolution of research (**Figure 8**). Duplicates of keywords such as “triglycerides” and “triglyceride” were merged by thesaurus. Clusters were significant, with a Q value of 0.3263, and reasonable with an S value of 0.6561. The cluster label was the most important keyword for each cluster. The most outstanding cluster was cluster #0 apolipoprotein B, followed by cluster #1 type 2 diabetes, cluster #2 insulin resistance, cluster #3 cardiovascular disease, cluster #4 physical activity, cluster #5 fatty acids, and cluster #6 lipid metabolism. Among the nodes of clusters, postprandial lipemia (n = 176, BC = 0.17, #0), type 2 diabetes (n = 358, BC = 0.47, #1), insulin resistance (n = 188, BC = 0.13, #2), metabolic syndrome (n = 152, BC = 0.12, #2), cardiovascular disease (n = 248, BC = 0.37, #3), lipid metabolism (n = 94, BC = 0.12, #6) acted as joints between clusters with high frequencies of co-citation and BC. Oxidative stress (n = 98, #4) and fatty acid (n = 56, #5) have also been exhaustively investigated. Apo B48 (n = 27, #0), one of Apo B transcripts, has particularly become important in research.

The top 20 keywords with citation bursts are shown in **Supplementary Figure S3B** and are also highlighted with red nodes in **Figure 8**. The keyword with the strongest citation burst was “systematic review” (strength = 5.82), followed by “gut microbiota” (strength = 5.44), and “oral fat tolerance test” (strength = 5.34). These were potential topics that were associated with transformative discoveries. The keywords, “remnant cholesterol”, “blood glucose”, “gut microbiota”, “systematic review”, “lipid profile”, “weight loss”, and “meal timing”, showed strong citation bursts until 2022.

## 4 Discussion

### 4.1 General information

A scientometric analysis and visualization were performed using studies on non-fasting lipid published from 2012 to 2022. The number of publications maintained a steady increase, with rapid citations. This implies that the non-fasting lipid research field has a bright future. Studies in this field were found to be relatively mature in the United States and the United Kingdom, with both productive publications and high citations per document. Although Denmark was the 11^th^ most productive country, the average citation times for each document was the highest, indicating that Danish papers are of a higher quality. In terms of publications, the outputs are burgeoning and booming in China, however, the citations per document are relatively low, suggesting that there is still a long way for Chinese researchers. There were strong collaborations among countries/regions regarding research in this field. Globally, the University of Copenhagen in Denmark is the most influential center in this field, and exhibited the most active connections with other institutions. Professor Børge G Nordestgaard, who is affiliated to the University of Copenhagen, made the most outstanding contribution to this field. The dual-map overlaps and subject category list suggest this is a multidisciplinary area. Clinical medicine and its sub-groups, nutrition and food science, biochemistry and molecular biology complement each other in this field. In terms of journals, *Nutrients* is the most popular journal, with the largest number of publications on non-fasting lipid. Among the most co-citation journals, *American Journal of Clinical Nutrition* ranked the first.

### 4.2 Emerging trends, hotspots, and frontiers

Co-citation reference networks reveal the research structure in the non-fasting lipid field. If a reference is co-cited by numerous publications, it is considered a basis in the field. The top 20 references with the strongest bursts and cluster transformation illuminated the trends of the field. Among the co-citation references, four papers had a high BC, indicating that potential structural variations or revolutionary points may occur at these nodes (3, 31–33). Through analysis of context in references, we concluded that cluster labels in **Figure 7** from #0 to #6 represent the following topics: #0 lipid-lowering strategies, #1 statin therapy, #2 high-fat meal, #3 metabolic response, #4 insulin resistance, #5 physical exercise, #6 fructose. The timeline view of co-cited keywords shows that hotspots in the non-fasting lipid area varied over time. Cluster analysis of keywords revealed the hot topics. Keywords with the strongest bursts represent alterations of frontiers. The #0 cluster label of co-occurrence keywords was Apo B, indicating that research related to Apo B recently received the most interest. Based on occurrence time, burst strength and maintenance period, “oral fat tolerance test”, “gut microbiota”, and “meal timing” keywords were the most noteworthy topics.

The co-cited references and keyword networks showed that research focus in this field has shifted from observational studies recognizing hazards of non-fasting lipid disorders in diverse diseases to investigating management methods and implementation of preventive strategies. Causal relationships among non-fasting dyslipidemia, CVD, and type 2 diabetes are still under investigation. The major aspects of management approaches include non-fasting lipid routine test and OFTT to identify potential postprandial dyslipidemia. Prevention strategies include lipid-lowering medications, lipid modification by anti-diabetic medications, and lifestyle interventions, such as physical exercise and diet changes. The association between gut microbiota and non-fasting lipid are being investigated.

#### 4.2.1 Observational evidence

Two prospective cohort studies published in 2007 are milestones in this field. One of them provided powerful evidence on the importance of non-fasting rather than fasting TG as the crucial risk factor for cardiovascular events (2). The other study, which showed that elevated non-fasting TG levels are associated with an increased risk of myocardial infarction, ischemic heart disease, and death acted as a junction for researches about #1 statin therapy, #2 high-fat meal, #3 metabolic response, #4 insulin resistance, and #6 fructose from 2012 to 2016 (3). Elevated non-fasting TG levels have also been shown to be a risk factor for ischemic stroke (4). However, due to the complexity of TG metabolism, it has not been established whether TG causes CVD. When TG levels are elevated, TRL, chylomicrons and very low-density lipoprotein (VLDL) contains more TG or increases in number of particles. In 2016, it was proven that elevated TRL, which cannot be fully reflected in a fasting sample, is a causal risk factor for inflammation, CVD and mortality (34). Recently, mendelian randomization analysis showed that suppressed Apo B levels reflect the benefits of reducing TG and LDL-C concentrations for a low risk of coronary heart disease, complied with genetic variants (38). Based on increasing evidence, routine Apo B assessments should be considered in clinical practice. A previous meta-analysis reporting that diabetes and fasting blood glucose are risk factors for various vascular diseases formed the bond in researches on #1 statin therapy, #2 high-fat meal, #3 metabolic responses, #4 insulin resistance, and #6 fructose from 2012 to 2017 (32). The research article reported that it was fructose and not glucose that induces insulin resistance and dyslipidemia, which led to researches on #6 fructose with #4 insulin-resistance and #3 metabolic response from 2015 to 2017 (33). Research heat on #6 fructose faded in 2018.

#### 4.2.2 Screening strategies

In the past decade, global guidelines and consensus have emphasized non-fasting lipid management. According to the 2011 European Society of Cardiology (ESC)/EAS guidelines for dyslipidemia management, high-density lipoprotein cholesterol (HDL-C), TC, Apo B, and Apo A1 levels can be assayed in non-fasting samples, whereas TG levels and LDL-C, which is calculated by the Friedewald formula should be assessed in fasting samples (39). The 2011 EAS guidelines for patients who achieved the LDL-C goal but still had high cardiovascular risks recommend that elevated TG, TRL and remnant cholesterol, and/or low HDL-C levels require further clinical management. The 2011 EAS guidelines also acted as the most influential bridge among researches on #4 insulin resistance, #2 high-fat meals, and #2 statin therapy from 2012 to 2019 (31). The 2013 American College of Cardiology/American Heart Association guidelines for cholesterol reduction to prevent atherosclerotic cardiovascular risk provided the non-fasting non-HDL-C and TG cut-off points for reappraisal. However, the guidelines preferred fasting lipid assessments (40). In 2016, the EAS/European Federation of Clinical Chemistry and Laboratory Medicine joint consensus provided a recommendation that non-fasting blood samples be used for routine screening of lipid profiles. Among the lipid profiles, TG, TC, and calculated concentrations of LDL-C, remnant cholesterol, and non-HDL-C are mostly affected by non-fasting status, whereas concentrations of HDL-C, Apo A1, Apo B, and lipoprotein(a) are not affected. Thus, the guidelines provided the cut-off levels for situations where fasting lipids are required to complement routine measurements of non-fasting lipid (35). The Canadian guidelines for dyslipidemia management also recommended non-fasting lipid and lipoprotein examinations during routine screening (12). The 2019 ESC/EAS guidelines showed that in terms of screening for prognostic outcomes of cardiovascular risks, high TG levels of 0.3 mmol/L (27 mg/dL) in non-fasting samples had equivalent clinical significance to fasting samples (15). However, fasting samples are recommended for patients with metabolic syndromes, diabetes, severe dyslipidemia, and follow-up for patients with hypertriglyceridemia (15, 41).

#### 4.2.3 Postprandial metabolic responses

Almost 40 years ago, Zilversmit hypothesized that atherogenesis might occur over a postprandial period when chylomicrons and/or remnants are elevated (42). Postprandial responses represent a typical non-fasting state (43). Based on available evidence, appropriate strategies should be developed to screen for and treat postprandial dysmetabolism to prevent CVD and diabetes (44). The OFTT is used to identify patients with postprandial lipidemia. However, this approach should be standardized. In 2011, experts established guidelines on non-fasting and postprandial TG levels, including classified concentrations, a practical project of OFTT, and treatment recommendations (16). However, evidence of elevated postprandial TG is not conclusive. In 2019, OFTT trials were summarized in a series of metabolic diseases and non-fasting TG is still considered as evidence for postprandial hypertriglyceridemia measurements (10). Implementable schemes and the benefits of fat overload have not been fully established. Metabolic responses of fat overload tests that had been adjusted to body weight versus standardized high-fat meals were compared in a randomized crossover study, suggesting that postprandial TG levels of healthy participants fluctuate in an acceptable scope with both interventions, supporting the feasibility of standardized meals in both scientific studies and clinical practice (45). Apo C3 reflects the risk of postprandial TG disorders (46). Instead of fat distribution, postprandial TG levels may reflect early fat metabolic disturbances (47). Chinese people with fasting TG levels in the range of 1.0 to 1.7 mmol/L may benefit most from OFTT to identify postprandial dyslipidemia (48). The triglyceride-insulin-glucose-glucagon-like peptide-1 model was built to assess glucose, insulin, and incretin responses following a high-fat meal (49). The OFTT can help patients identify insulin sensitivity impairments, insulin resistance, beta-cell dysfunctions and secretion disorders of gut hormones, including glucose-dependent insulinotropic polypeptides and glucagon-like peptide-1 (21, 50).

Apo B is considered to be an indicator of atherogenic lipoproteins. Particularly, Apo B48 is an emerging hotspot that is increasingly being studied. After a meal, TGs are packaged into chylomicrons particles and transported to various tissues. Apo B48 which is synthesized by the intestines is a unique biomarker of postprandial intestinal chylomicron metabolites, which indicates the absorption of TG (51). Observational studies have reported that elevated fasting Apo B48 remnants associated with fat intolerance can predict postprandial Apo B48 disorder and increase the risk of CVD in adolescents with obesity (52). Postprandial Apo B48 contributes to subclinical atherosclerosis in patients with rheumatoid arthritis (53). The intestinal FGF15/19-SHP-TFEB pathway is activated after a meal, which mediates lipophagy and regulates postprandial TG and chylomicron via Apo B48. This study revealed a novel target for postprandial dyslipidemia treatment (54). Statin therapy can prevent the production of Apo B48 in intestines and promote the catabolism of Apo B48 to reduce postprandial TG synthesis (55). Semaglutide can decrease the production of postprandial Apo B48 levels (56). T2DM patients treated with liraglutide showed decreased levels of Apo B48, swelling of chylomicrons, reduced chylomicrons and remnant formation (57). Diet composition can also affect Apo B48 levels. A comparison of two common saturated fat of mixed meals, higher palmitic-acid intake found lower Apo B48 levels than higher stearic-acid intake (58).

#### 4.2.4 Medication regimens

Evidence from many studies has confirmed that LDL-C causes atherosclerotic cardiovascular disease (37). The guidelines show that statin therapy should be the preferred treatment to reduce LDL-C levels compared with other lipids-lowering strategies because it can prevent and improve the prognosis of cardiovascular events (7, 12, 15). Elevated TG cannot be underestimated, especially in a non-fasting state (59).The results of the IMPROVE-IT trial revealed that the combination of ezetimibe and statin therapy was more effective and did not cause adverse effects (60). In 2017, evolocumab combined with statin therapy was found to reduce the risk of cardiovascular events in the FOURIER trial (61). In 2019, the results of REDUCE-IT trial showed that icosapent ethyl decreased the risk of ischemic events among patients with hypertriglyceridemia receiving statin therapy (36). As shown in the co-citation reference networks from 2019 to 2022, #1 statin therapy was changed to #0 lipid-lowering strategies, suggesting that other drugs for dyslipidemia treatment are under investigation.

For the cluster #4 insulin resistance, the main research topic was the cardiovascular protective effects of anti-diabetic medications due to improved postprandial lipids metabolism, particularly, incretin-based drugs including dipeptidyl peptidase-4 inhibitors, glucagon-like peptide-1 receptor agonists (62, 63). Some of these medications have been proven to reduce TRL significantly (57, 64).

#### 4.2.5 Lifestyle interventions

Lifestyle interventions included physical exercise and chrononutrition. Research on the application of #5 physical exercise to reduce postprandial lipidemia increased in the 2013-2014 and 2018-2020 period. From the cluster analysis of keywords, we found that oxidative stress was highly linked to physical exercise. Endurance exercise can have a beneficial impact on postprandial TG levels, however, it does not seem to affect markers of oxidative stress (65). Regular physical exercise even walking can improve postprandial lipidemia and blood glucose (66, 67). Aerobic exercise has similar effects with statin therapy in terms of reducing postprandial TG levels as reported in a previous meta-analysis (68). In 2017, scientists won the Nobel Prize for elucidating the biological clock. Since then, research on the circadian variation of lipids has experienced bursts into hotspots. Meal timing strongly affects postprandial lipid profiles (69). Nighttime food intake may also increase the risk of dyslipidemia and hence potential of CVD due to postprandial TG metabolic dysfunction (70). Feeding time restricted within eight hours for five days can increase the fat oxidation rate in both fasting and non-fasting state, but cannot affect postprandial lipemia (71).

#### 4.2.6 Gut microbiota

Variation in intestinal microbiota composition and drugs targeted at gut microbiota have been investigated from animals to human beings (9). In animal studies, a high-fat/high-fructose diet causes gut microbial alteration in hamsters leading to metabolic syndrome (24). Extracts of fermented green tea were reported to reduce high-fat diet-induced hypertriglyceridemia in hamsters by decreasing the proportion of phylum Firmicutes in gut microbiota (72). In human studies, the PREMOTE trial showed that the combination of berberine and probiotic improved postprandial dyslipidemia among T2DM patients, providing a novel therapy targeting gut microbiota (73). Importantly, the PREDICT 1 trial built a machine-learning model to evaluate metabolic responses to food intake involving meal nutrients, meal timing, physical exercise, diurnal rhythm, and gut microbiota. This model can function as an algorithmic tool that allows for the accurate customization of nutritional strategies in the treatment of illnesses (25).

### 4.3 Advantages and limitations

This is the first bibliometric analysis to provide a credible account of the current state of research on non-fasting lipids. The findings of this study are expected to shed light on new directions and reveal ideas for further investigations. However, there are still some limitations. First, owing to the nature of the bibliometric software, we only analyzed data from the WoSCC (SCI-E) database. Second, only English-language papers were analyzed. Therefore, this study may not fully represent the entire spectrum of this field. Third, some new ideas may have been overlooked because the most recently published papers often have lower citations.

## 5 Conclusions

This bibliometric analysis of non-fasting lipid from 2012 to 2022 was conducted to visualize trends, hotspots and frontiers. The study of lipids in a non-fasting state is highly important in various fields including nutrition, endocrinology, cardiology, pharmacology, molecular biology, and sports medicine. In this field, researchers have switched from observing hazards of non-fasting dyslipidemia to elucidating the causal relationship of non-fasting dyslipidemia in diseases, especially cardiovascular diseases and diabetes. The guidelines and expert consensus are constantly being updated regarding the recommendations for non-fasting lipid assessment. The investigation of standardized OFTT to identify potential postprandial dyslipidemia is ongoing. The lipid-lowering strategies, statin therapy, high-fat meal, insulin resistance, physical exercise, and fructose are hotspots. Identification of optimal lipid-lowering strategies should be intensified with a focus on lipid-lowering drugs, lipid-reducing effects of anti-diabetic drugs (especially incretin-based drugs), physical exercise and nutrition therapies. Apo B48, gut microbiota, and chrononutrition are the frontiers in the research field of non-fasting lipid.

## Data availability statement

The datasets presented in this study can be found in online repositories. The names of the repository/repositories and accession number(s) can be found in the article/**Supplementary Material**.

## Author contributions

YH: Conceptualization, Methodology, Formal analysis, Data Curation, Visualization, Writing - Original Draft. ZA: Validation, Formal analysis, Data Curation. XH and YG: Validation, Supervision. GS: Conceptualization, Supervision, Writing - Review and Editing. All authors contributed to the article and approved the submitted version.
