# Supplementary material for: A bibliometric analysis and visualization of literature on non-fasting lipid research from 2012 to 2022
Source: Front Endocrinol (Lausanne). 2023 Apr 19;14:1136048. doi: 10.3389/fendo.2023.1136048 (PMC10154597; doi:10.3389/fendo.2023.1136048)
Supplement: Supplementary file 1 [file DataSheet_1.zip › Supplementary_Material/Supplementary_Material.docx]

Supplementary Material

A Bibliometric Analysis and Visualization of Literature on Non-fasting Lipid Research from 2012 to 2022

Yilin Hou, Zehua An, Xiaoyu Hou, Yunpeng Guan and Guangyao Song^*^

*** Correspondence:**

Guangyao Song

sguangyao2@163.com

# Supplementary Data

This study's raw data can be found in the raw data file.

# Supplementary Figures


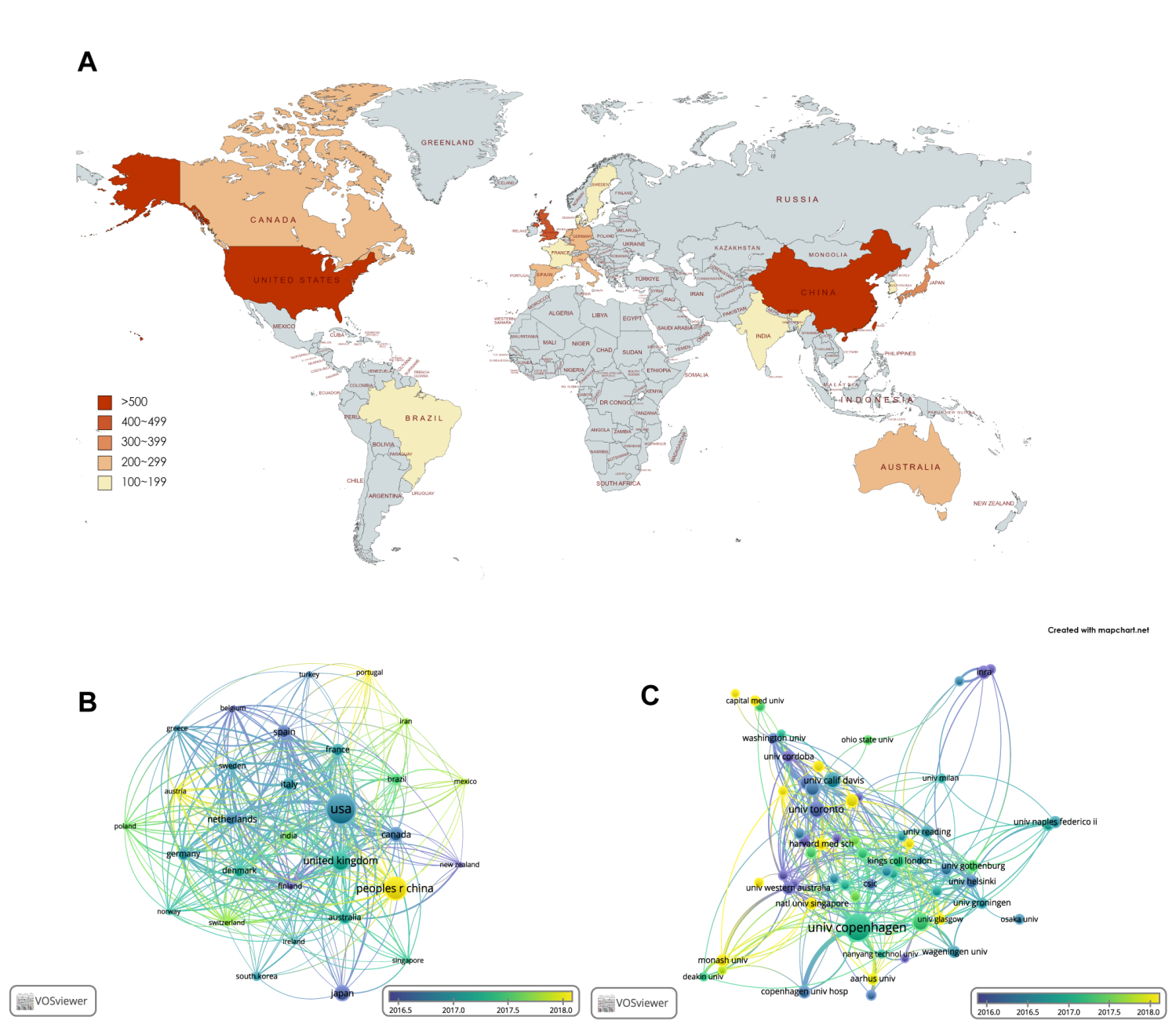


**Supplementary Figure S1** Visualization of countries and institutions. **(A)** World map of publication distribution in countries. **(B)** Collaboration network among countries. **(C)** Collaboration network among institutions.

**
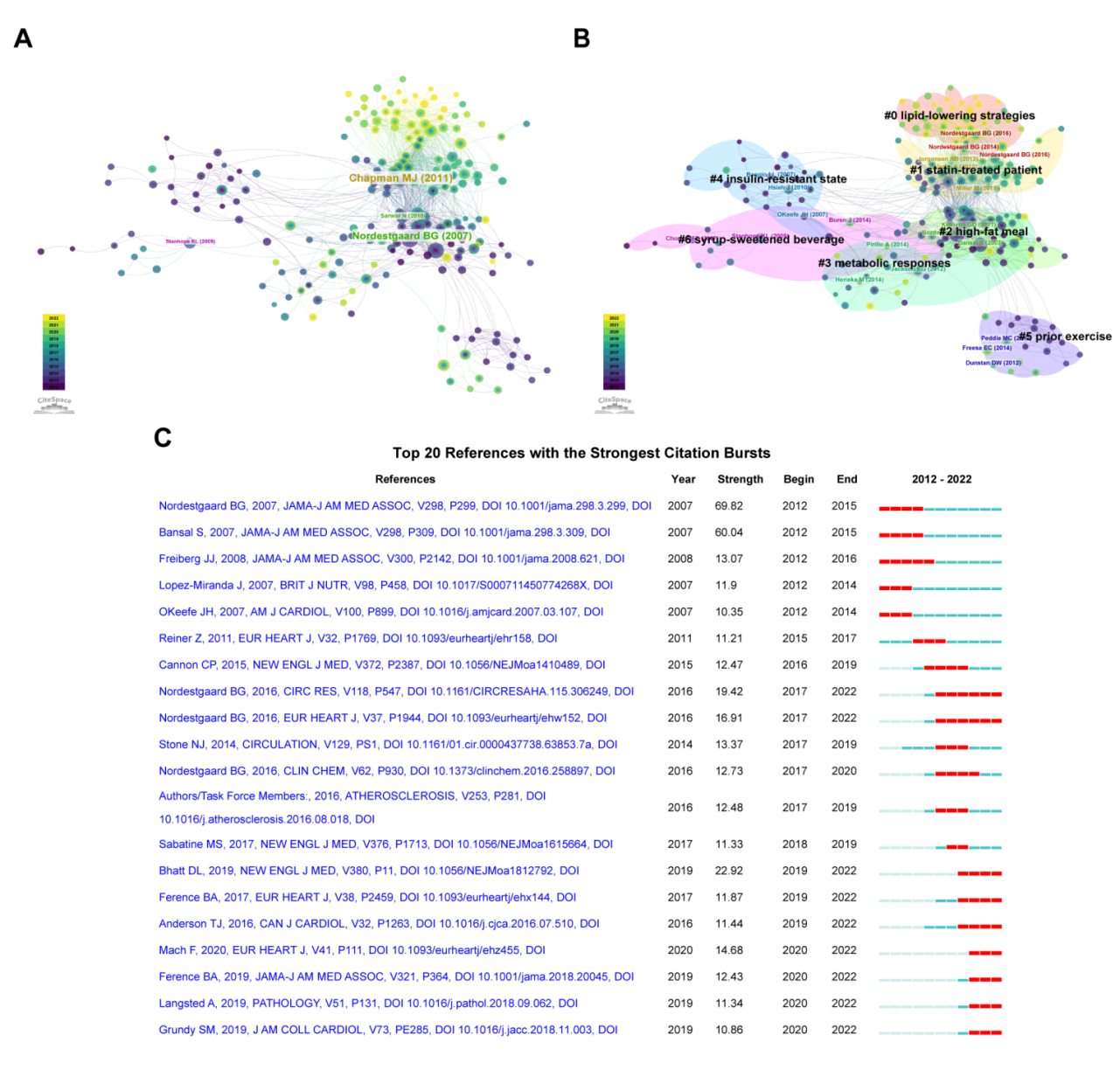
**

**Supplementary Figure S2** Co-citation references visualization. **(A)** Co-citation references network. The publications with highest betweenness centrality were circled by purple round and labelled by author name and publication year. **(B)** Cluster analysis of co-citation network. The top three co-citation references of each cluster labelled by author name and publication year. **(C)** Top 20 references with the strongest citation bursts.


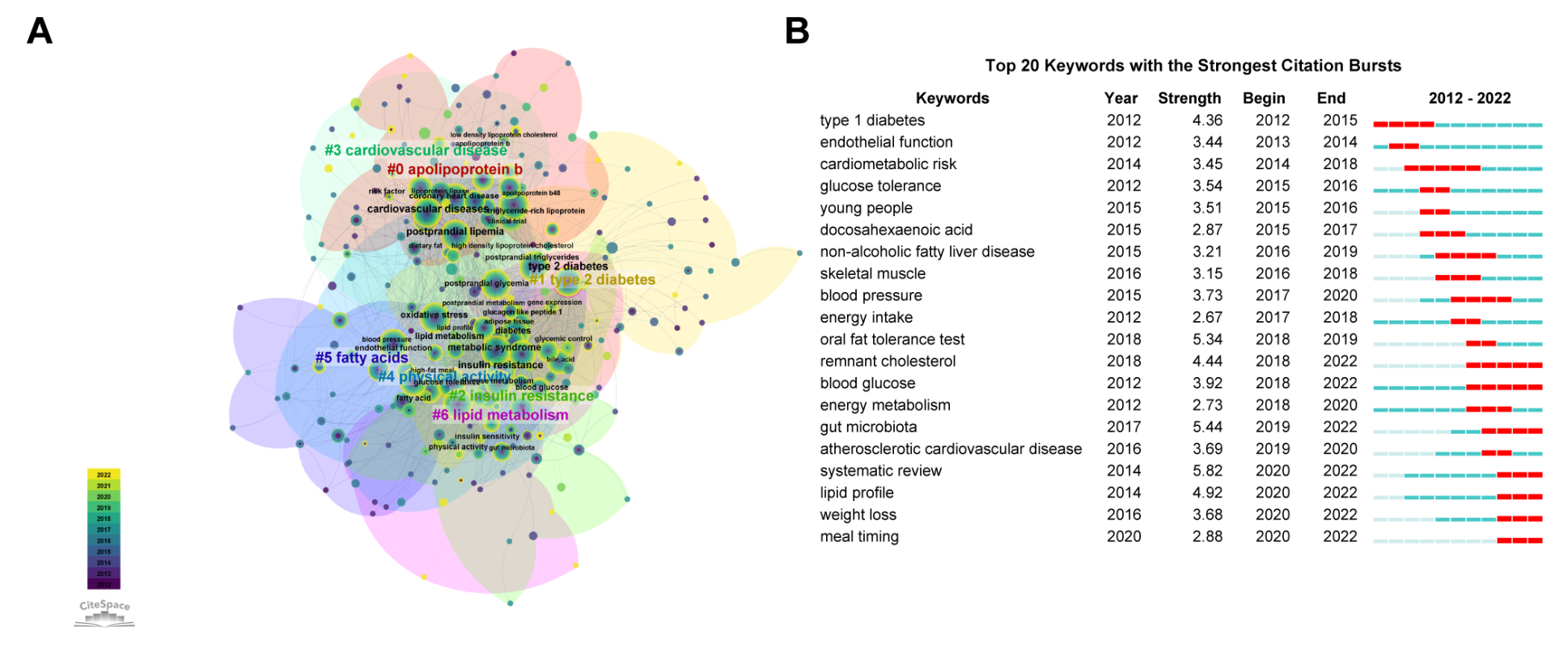


**Supplementary Figure S3** Keywords Visualization. **(A)** Cluster analysis of co-citation keywords. **(B)** Top 20 keywords with the strongest citation bursts.
